# Defect–Coating–Wavelength Coupling Effects on Nano-Scale Electric Field Modulation in Fused Silica Under Multi-Wavelength Irradiation

**DOI:** 10.3390/nano15211626

**Published:** 2025-10-25

**Authors:** Hongbing Cao, Xing Peng, Feng Shi, Xinjie Zhao

**Affiliations:** 1 College of Intelligence Science and Technology, National University of Defense Technology, Changsha 410073, China; hb_c@nudt.edu.cn (H.C.); shifeng@nudt.edu.cn (F.S.);; 2 National Key Laboratory of Equipment State Sensing and Smart Support, Changsha 410073, China; 3 Hunan Provincial Key Laboratory of Ultra-Precision Machining Technology, Changsha 410073, China

**Keywords:** fused silica, FDTD, anti-reflection coatings, multi-wavelength laser

## Abstract

Fused silica optical components with antireflection (AR) coatings are key components in high-power laser systems. However, their reliability is severely challenged by multi-wavelength irradiation and the presence of unavoidable matrix surface defects. To investigate the coupling effects of electric field modulation between multi-wavelength irradiation, AR coating layers, and defects in AR-coated fused silica, this paper uses the finite-difference time-domain (FDTD) method to simulate the nanoscale electric field intensity distribution in fused silica coated with a double-layer AR coating at three different design wavelengths using multi-wavelength lasers. The effects of electric field coupling between the coating layers and defects are analyzed for three representative scratch geometries. The results show that when the incident wavelength matches the AR design wavelength, the interface field is effectively suppressed, resulting in a smoother field distribution and localized hot spots. Conversely, mismatched wavelengths induce severe field distortion, producing multiple hot spots and lateral interference fringes. Wide, shallow scratches are particularly sensitive to wavelength mismatch, with a 532 nm AR coating exhibiting a global maximum enhancement factor of 1.63442 for 355 nm incident light. These findings highlight the coupling effects of scratch geometry, AR coating dispersion, and laser wavelength on electric field modulation. This research provides valuable insights for optimizing antireflection coatings and improving defect tolerance in multi-wavelength laser applications, helping to improve the reliability of high-power laser systems.

## 1. Introduction

High-power laser systems are widely used in precision machining, laser nuclear fusion, defense equipment, and space technology. Their core optical components often need to work stably for a long time under extreme light intensity conditions [1]. Fused silica is widely used in components such as windows, lenses, and prisms in high-power laser systems due to its excellent transmission performance, chemical stability, and mechanical strength [2]. In order to reduce interface reflection loss and improve transmission efficiency, the surface of fused silica is usually coated with an anti-reflection coating (AR coating) optimized for a specific wavelength [3]. However, in actual working environments, optical components are not only irradiated with the designed wavelength but are also often irradiated with light of other wavelengths, such as second harmonics, third harmonics, scattered light, or system crosstalk light [4,5]. In this case, the incidence of non-designed wavelengths may lead to local interference effects in the coating layer and distortion of the electric field distribution, thereby generating local electric field enhancement and accelerating the damage process of the component [6]. In addition, optical components will inevitably introduce surface or subsurface defects such as scratches, pits, particles and microcracks during manufacturing, transportation and use [7]. These defects will not only destroy the uniformity of beam propagation but may also induce electric field concentration in the defect area, forming so-called “hot spots”, significantly reducing the damage threshold of the component [8]. Studies have shown that even under ideal coating design conditions, local defects can cause the electric field enhancement factor to exceed 2–5 times, thus becoming an important trigger point for early failure of optical components [9,10]. Therefore, when evaluating the service performance of fused silica optical components, it is necessary to consider both the multi-wavelength irradiation effect and the modulation effect of defects on the electric field distribution. FDTD is widely used in the study of optical coatings, defects and damage mechanisms by solving Maxwell’s equations [11].

Ling et al. used the finite difference time domain method to study the electric field concentration effect caused by defects in optical thin coatings, revealing the sensitivity of geometric scale to the amplification of local field strength [10]. Zhang et al. further combined strong field electron dynamics with FDTD and proposed a new approach to predict dielectric coating damage, emphasizing the direct relationship between “field distribution-damage” [12]. Similarly, Cheng et al. verified through artificial defect experiments that high field hot spots induced by nodule defects are the key mechanism for the early initiation of damage, and the results are highly consistent with the numerical simulation results [13]. In terms of anti-reflection coatings, studies have shown that they can effectively reduce interface reflection and smooth the electric field distribution at the design wavelength. Shao et al. prepared subwavelength anti-reflection structures on the surface of fused quartz, demonstrating the feasibility of suppressing Fresnel reflection and improving transmission [14]. Astrauskytė et al. used the atomic layer deposition (ALD) process to prepare anti-reflection coatings, and the measured reflectivity was significantly reduced from 3.3% to 0.1%, which directly reflects the inhibitory effect of the optimized coating layer on the interface field. At the same time, it was found that this improvement has limitations at non-design wavelengths [15]. Tsibidis et al. studied the response of Si-based AR coatings under mid-infrared femtosecond laser irradiation and found that wavelength mismatch would lead to enhanced and distorted electric field stripes within the coating layer [16]. Gallais and Commandré also showed through multi-band laser experiments that when the incident wavelength deviates from the coating design value, the laser damage threshold of the material and coating decreases significantly [5]. In terms of experimental diagnosis and mechanism analysis, Liu et al. [17] used pump-probe microscopy to reveal the plasma generation, shock wave and crack evolution process of fused quartz under ultrafast laser irradiation, providing a dynamic verification method for numerical simulation results. Yudin et al. pointed out that silicon agglomerates in interference AR coatings can significantly reduce laser-induced damage threshold (LIDT), indicating that microscopic inhomogeneity of the coating layer may also cause field distortion and failure [18]. Li et al. proposed a method to enhance the laser damage resistance of fused silica optical devices by coating the back of the optical element with a polyvinyl alcohol (PVA) film. Using FDTD simulation, they analyzed the effect of the PVA coating on the electric field distribution and found that a PVA coating of appropriate thickness could shift the maximum electric field intensity from the back to the coating-air interface, thereby improving the laser damage threshold [19]. Gao et al. investigated the propagation characteristics of point defects in SiO_2_/Al_2_O_3_ antireflection coatings and found that point defect propagation induced by multi-pulse laser irradiation was the primary cause of AR film performance degradation [20]. Du et al. analyzed the effect of a grating structure on the fused silica surface on the electric field distribution using FDTD simulation and found that the temperature distribution caused by the electric field distribution within the grating may be the primary factor causing damage [21]. Furthermore, Shan et al. and Hobbs et al. studied the performance of high-reflective (HR) coatings and nano-AR structures under high-power lasers, demonstrating the intrinsic connection between electric field enhancement and coating damage evolution, and proposed the potential of coating-free nanostructures to be more robust in multi-wavelength environments [22,23]. Recently, Shao et al. effectively suppressed secondary defects on the surface of fused quartz by oxygen-assisted etching, increasing the damage threshold by more than 120%, emphasizing the importance of synergy between process optimization and coating design [24].

For example, a tripled frequency Nd:YAG laser can generate multi-wavelength outputs of 355 nm, 532 nm and 1064 nm. These lasers inevitably interact in complex systems and irradiate optical components together [15]. Under such conditions, a single-wavelength optimized anti-reflection coating may exhibit abnormal electric field distribution characteristics at non-design wavelengths, especially in the presence of defects, which is more likely to induce strong local electric field enhancement. Therefore, it is necessary to systematically study the electric field distribution of fused quartz surfaces coated with different wavelength AR coatings and defects under multi-wavelength irradiation. This paper uses the FDTD method to simulate the electric field distribution characteristics of fused quartz surfaces coated with different wavelength AR coatings at three typical laser wavelengths: 355 nm, 532 nm, and 1064 nm. Furthermore, several typical scratch defect structures are introduced to explore the coupling between multi-wavelength irradiation and defect effects, revealing their influence on the electric field enhancement and hotspot distribution.

## 2. Model and Methods

**Figure 1:**
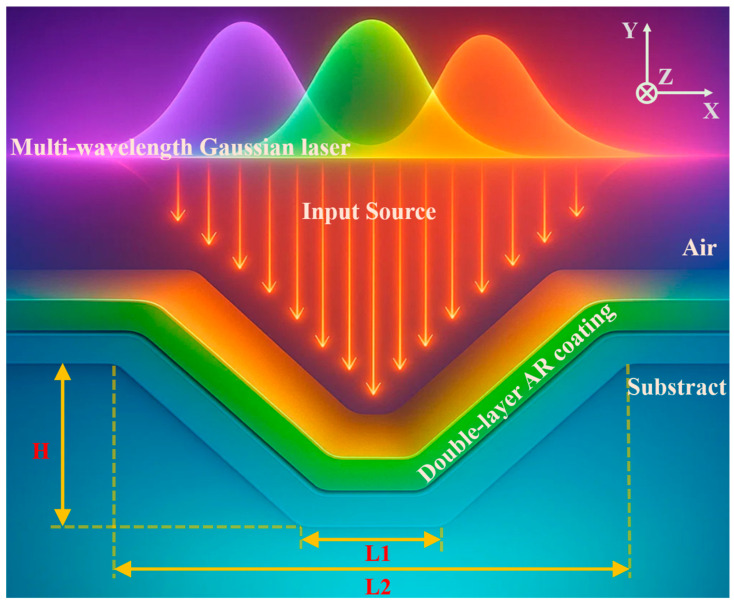
Schematic diagram of the simulated irradiated scratch model. The upper part of the figure represents the Gaussian incident laser source of 355 nm, 532 nm, and 1064 nm.

This study used a two-dimensional FDTD method to numerically simulate an air/double-layer AR coating/fused silica substrate system. Antireflection coatings are typically fabricated using methods such as chemical vapor deposition (CVD), sputtering, and ALD. These methods allow for precise control of coating thickness and material properties. ALD, by alternately introducing two precursor gases and depositing one atomic layer vertically on the component surface, allows for precise control of film thickness and uniformity, making it a common method for fabricating high-precision antireflection coatings. Therefore, the model assumes that the thickness of the double-layer antireflection coating remains constant in the deposition direction, which is closer to the actual situation. The overall geometry is shown in Figure 1. The incident laser, a Gaussian source with selectable wavelengths of 355 nm, 532 nm, and 1064 nm, was incident perpendicularly from the air side, sequentially passing through the double-layer coating system and impacting the substrate surface. To simulate typical surface damage precursors, scratches were selected as a representative defect type.

This work uses the FDTD to directly solve the Maxwell equations in the time domain. When magnetic dispersion and magnetic loss are ignored, the medium satisfies:

(1) $$\nabla \times E=-\mu \frac{\partial H}{\partial t},\nabla \times H=-\epsilon \frac{\partial E}{\partial t}+\sigma E$$

Scratches are one of the most common surface damage features of fused silica components during manufacturing, handling, and service. Compared to defects such as pits, dents, and microcracks, scratches tend to have larger length scales and a higher probability of occurrence. Their geometric morphology (sharp corners and grooves) easily induces localized electric field distortion and hotspot effects [25]. Therefore, studying scratches as a typical defect better reflects the main risk factors in actual engineering applications.

The three sets of double-layer AR coating designs studied in this paper are designed for three typical laser wavelengths: 355 nm, 532 nm, and 1064 nm. They all adopt the quarter-wavelength thickness design principle, that is, the coating thickness satisfies Equation (2).

(2) $$d=\frac{\lambda }{4n}$$

The specific coating material combination is as follows: 355 nm AR coating is HfO_2_/SiO_2_, 532 nm and 1064 nm AR coatings are: Ta_2_O_5_/SiO_2_.

The refractive index of the fused silica substrate adopts the Sellmeier equation given by Malitson, as shown in Equation (3) [26].

(3) $$n^{2}\left(\lambda \right)=1+\frac{0.6961663\lambda^{2}}{\lambda^{2}-0.0684043^{2}}+\frac{0.4079426\lambda^{2}}{\lambda^{2}-0.1162414^{2}}+\frac{0.8974794\lambda^{2}}{\lambda^{2}-9.896161^{2}}$$

The dispersion data of other materials are taken from the RefractiveIndex. INFO database [27,28].

In the visible and near-infrared bands, the extinction coefficients of these materials are extremely low, which is consistent with experimental data [29]. The specific parameters are listed in Table 1.

To ensure the consistency of comparison and the normalization of results, the incident field amplitude of the three wavelength lasers is set to a simple harmonic wave of 1 V/m:.

(4) $$E_{z}^{inc}\left(x,t\right)=E_{0}Re\left\{e^{i(kx-\omega t)}\right\},\;\;\;\;\;\;\;H_{y}^{inc}\left(x,t\right)=\frac{E_{0}}{\eta_{0}}Re\left\{e^{i(kx-\omega t)}\right\}$$

where η0=μ0∈0 is the free space impedance.

Since the electric field and the incident amplitude are linearly related in the Maxwell framework, using the same amplitude will not affect the universality of the conclusions. The laser incident form is Gaussian, and the beam waist radius is 100 μm. Considering that scratch defects are typically much longer in length than in depth, a two-dimensional model can approximate their cross-sectional characteristics. TE polarization is chosen to effectively capture the electric field concentration effects at scratch corners and sidewalls without the complexity of three-dimensional calculations:

(5) $$E=\left(0,0,E_{z}\right)\;\;,\;\;\;\;H=(H_{x},H_{y},0)$$

To ensure that the monitor can effectively reflect the electric field enhancement distribution under incident laser light of different wavelengths, the simulation bandwidth is set to ±10 nm of the center wavelength. The simulation environment temperature is set to 300 K, the total simulation time is capped at 1 ns, and an automatic termination condition is set: the simulation automatically ends when the overall field strength decays below 10^−5^, balancing accuracy and efficiency. Perfectly matched layer (PML) boundary conditions are applied around the computational domain to effectively absorb the outgoing wave and prevent scattering from affecting the near-field distribution. The mesh is locally refined at the interface between the defect sidewall and the coating layer to accurately capture local field variations.

## 3. Calculation and Analysis

### 3.1. Multi-Wavelength Laser Irradiation of Defect-Free Fused Silica Coatings

**Figure 2:**
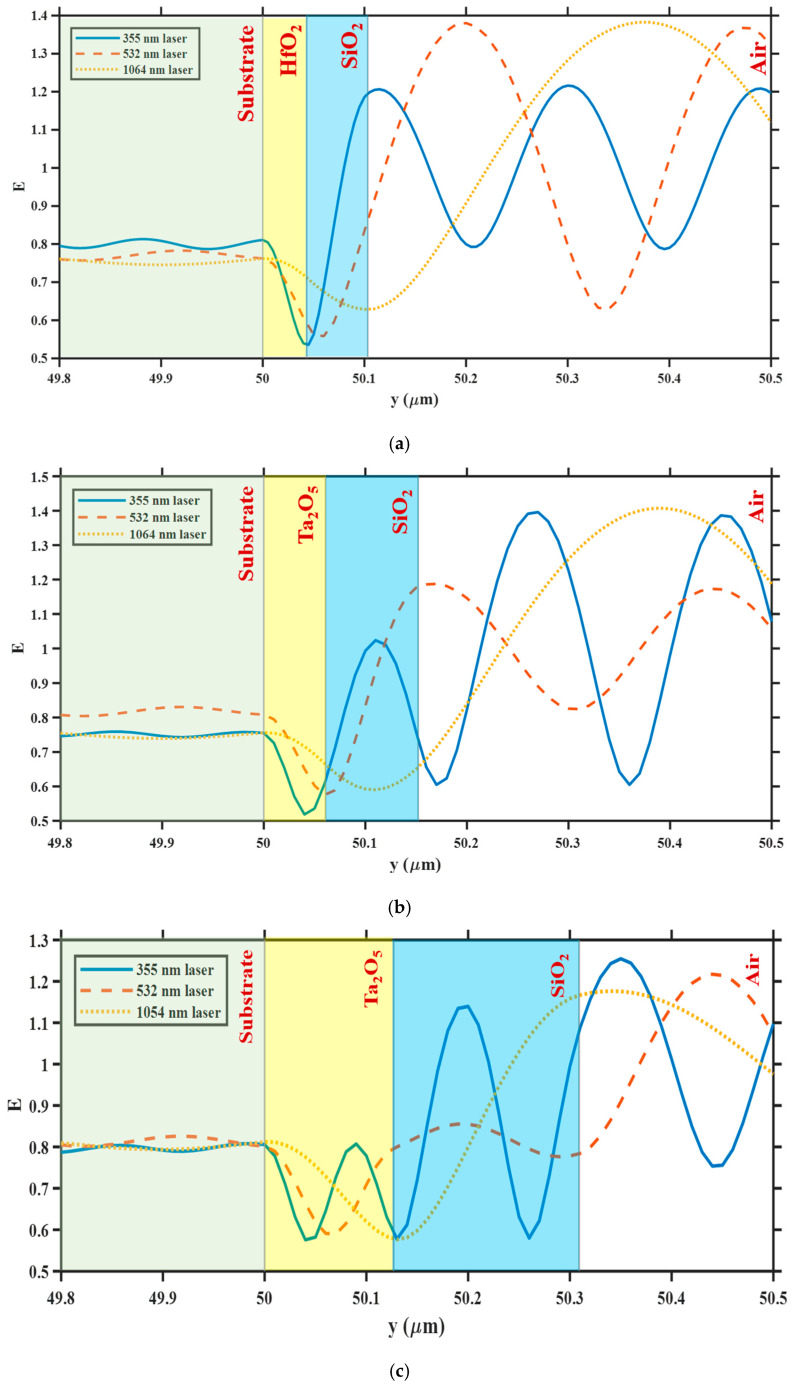
Electric field intensity distributions at the substrate–coating–air interfaces for (**a**) the 355 nm AR coating (HfO_2_/SiO_2_), (**b**) the 532 nm AR coating (Ta_2_O_5_/SiO_2_), and (**c**) the 1064 nm AR coating (Ta_2_O_5_/SiO_2_) under irradiation by 355 nm, 532 nm, and 1064 nm lasers.

To analyze the variation in electric field intensity of different AR coatings on the fused quartz surface under multi-wavelength laser irradiation, this section first considers the defect-free condition. Corresponding double-layer AR coating systems were constructed at design wavelengths of 355 nm, 532 nm, and 1064 nm and independently irradiated with three monochromatic laser sources. Figure 2 shows the electric field intensity distribution at the substrate-coating-air interface.

The results show that when the incident laser wavelength matches the design wavelength of the coating, the interface electric field is significantly suppressed. For example, when a 355 nm laser is incident on a 355 nm AR coating, the electric field intensity rapidly decays to approximately 0.5 V/m at the interface between the two layers after entering the coating from air. The field intensity at the coating-substrate interface is approximately 20% weaker than that on the air side, and remains at a low level after entering the substrate, with a significantly reduced amplitude. Similarly, when 532 nm and 1064 nm lasers are incident on the corresponding AR coatings, the interface electric field is weakened by approximately 19% and 20%, respectively, at the target wavelengths, significantly exceeding the performance at non-design wavelengths. This demonstrates that the quarter-wavelength AR coating effectively achieves reflection cancelation at the target wavelength, reducing the localized interface electric field intensity and forming a “low-field transmission” channel.

In contrast, when the coating is irradiated with non-design wavelengths, the interface electric field distribution becomes significantly distorted. For example, when a 355 nm laser irradiates a 532 nm coating, the electric field initially weakens upon entering the coating, but rebounds at the coating-substrate interface, ultimately reaching an intensity close to that of a 1064 nm wavelength. Conversely, when a 532 nm laser irradiates a 1064 nm coating, the wavelength within the coating is significantly distorted, with the peak significantly weakened, ultimately reaching an intensity close to that of the other two wavelengths. Notably, the electric field intensity of the 532 nm laser incident on the substrate after passing through the 532 nm AR coating is significantly higher than that of the other two lasers, while in other cases, the intensity is relatively similar. These results suggest that a coating optimized for a single wavelength can induce anomalous local field distributions when exposed to multiple wavelengths, reducing the AR effect and potentially forming potential hotspots at the interface.

**Figure 3:**
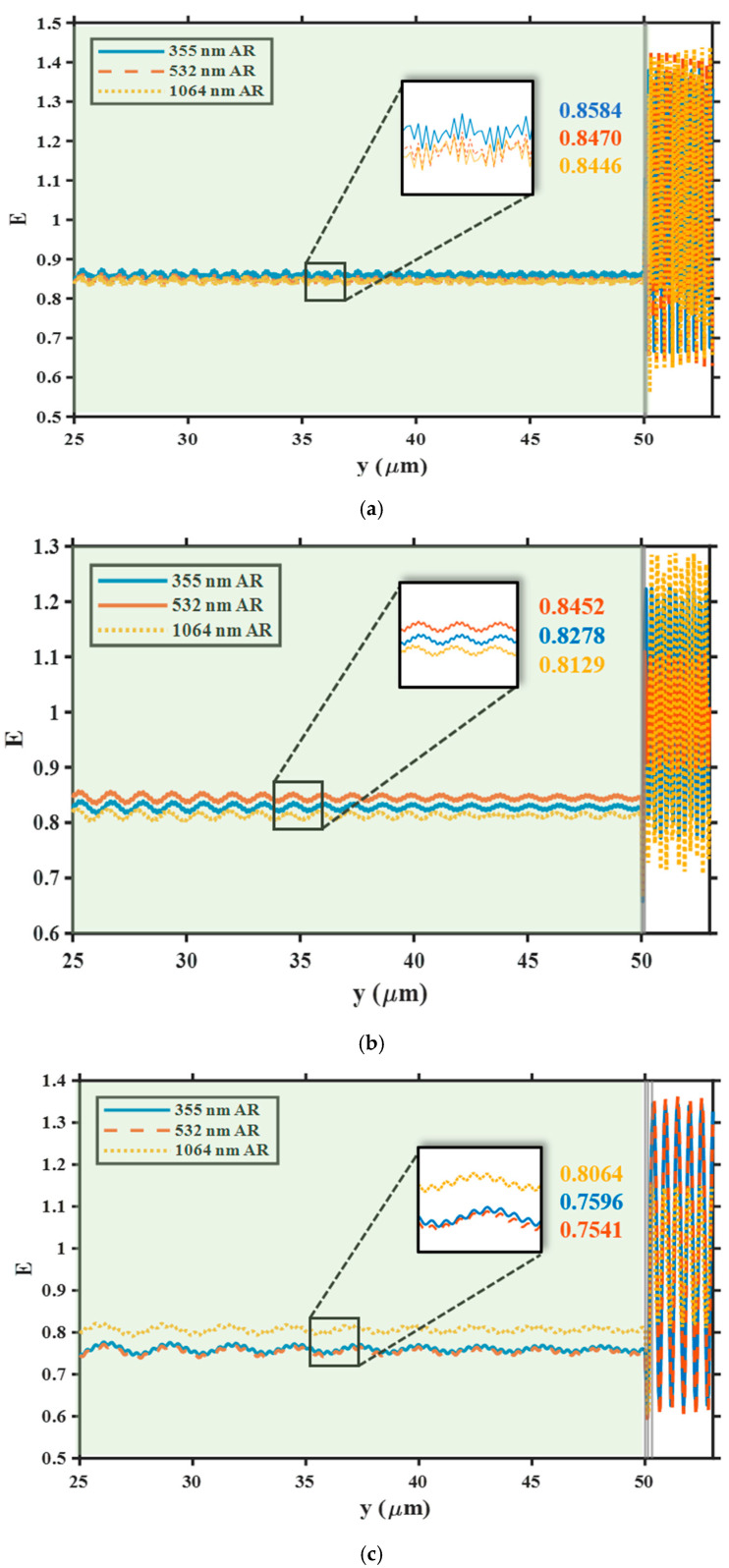
Comparison of electric field intensity distributions inside fused silica substrates when (**a**) 355 nm, (**b**) 532 nm, and (**c**) 1064 nm lasers irradiate all three AR coatings. Insets denote the average field intensities in each case.

Further results are shown in Figure 3, which shows the substrate electric field distribution and average electric field intensity when a single wavelength laser is applied to three different AR coatings. The average electric field intensity under the 355 nm z AR coating is the highest (0.8584 V/m), which is approximately 1.3% higher than that under the 532 nm coating and 1.6% higher than that under the 1064 nm coating (0.8446 V/m). The electric field Intensity of the 355 nm laser after passing through the 532 nm and 1064 nm AR coatings differs by approximately 0.3%. Under 532 nm laser irradiation, the average electric field strength after the 532 nm AR coating was 0.8452, an increase of approximately 2% compared to the 355 nm coating (0.8278 V/m) and approximately 4% compared to the 1064 nm coating (0.8129 V/m), a significant difference. Under 1064 nm laser irradiation, the 1064 nm coating exhibited the highest average electric field (0.8064 V/m), while the average electric fields of the 532 and 355 coatings were very close, with a difference of less than 1%. These results indicate that long-wavelength coatings significantly reduce the substrate electric field at their design wavelengths, with similar reduction effects at shorter wavelengths than their design wavelengths. Short-wavelength coatings achieve the highest average electric field strength within the substrate, but their reduction effect at longer wavelengths outside their design wavelengths is limited and close to that of their design wavelengths.

Overall, the dual-layer AR coatings at the three designed wavelengths all demonstrate excellent suppression effectiveness at their respective matching wavelengths, effectively reducing electric field reflection and localized enhancement at the air-coating-substrate interface, and smoothing the electric field distribution within the substrate. However, when the incident laser is mismatched with the design wavelength of the coating, obvious interference fringes and electric field distortion appear inside the coating, and the average electric field intensity within the substrate increases with the degree of mismatch, indicating potential risks under multi-wavelength irradiation. In addition, the overall amplitude of the electric field decreases significantly after entering the high-refractive-index fused silica substrate, which is mainly due to the wave impedance matching effect caused by the refractive index difference between air and the substrate. This shows that although the anti-reflection coating can effectively reduce the interface field strength at the design wavelength, its regulatory effect will be limited in a complex multi-wavelength environment. Irradiation with non-design wavelengths may induce local electric field enhancement within the coating and accelerate the damage process.

### 3.2. Influence of Scratch Depth and Width on Electric Field Modulation

**Figure 4:**
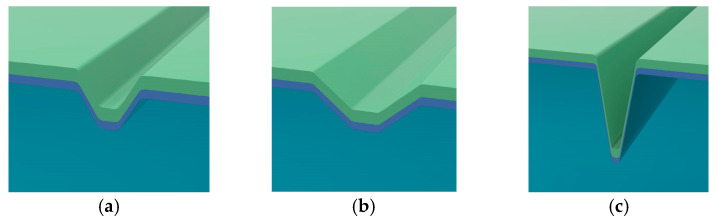
Schematic models of three types of scratch-induced defect.

**Figure 5:**
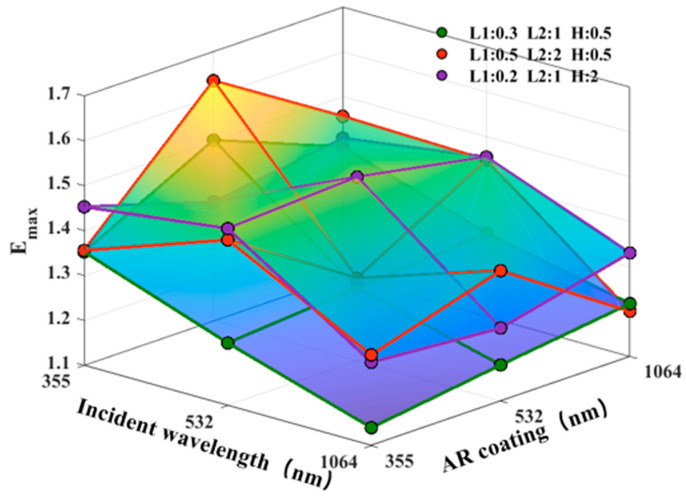
*E*_max_ under multi-wavelength laser irradiation with different AR coatings.

To further explore the effects of typical defects on the electric field distribution on the fused silica surface under multi-wavelength laser irradiation, this section establishes three representative scratch geometry models, as shown in Figure 4: (a) an inverted trapezoidal defect (L1 = 0.3 μm, L2 = 1 μm, H = 0.5 μm), (b) a relatively wide and shallow scratch defect (L1 = 0.5 μm, L2 = 2 μm, H = 0.5 μm), and (c) a deep and narrow scratch defect (L1 = 0.2 μm, L2 = 1 μm, H = 2 μm). To better reflect actual conditions, a 0.1 μm filet was used at the junction. Three double-layer AR coatings optimized for 355 nm, 532 nm, and 1064 nm wavelengths were deposited on the defect surface. FDTD simulations were performed using the corresponding three laser wavelengths, resulting in the maximum electric field intensity *E*_max_ for 27 different conditions, as shown in Table 2 and Figure 5.

For ease of presentation, the three typical scratch defect geometries are denoted as follows:

G1: L1 = 0.3, L2 = 1, H = 0.5; G2: L1 = 0.5, L2 = 2, H = 0.5; G3: L1 = 0.2, L2 = 1, H = 2.

From the above data, we can see that the global maximum of the 27 data points is 1.63442, corresponding to the combination of G2 at 355 nm incident light and 532 nm AR. The global minimum is 1.13983, corresponding to the combination of G1 at 1064 nm incident light and 355 nm AR. The difference between the two is 0.49459 (approximately 43.4%). From an average perspective of incident wavelength, the 355 nm wavelength has the highest average *E*_max_ (1.435), followed by the 532 nm wavelength (1.383), and the 1064 nm wavelength has the lowest (1.257), indicating that shorter wavelengths are more likely to trigger field enhancement near defects. From an AR design perspective, the 532 nm AR has the highest overall average *E*_max_ (1.377), exceeding both the 355 nm AR (1.343) and the 1064 nm AR (1.354). This suggests that under multi-wavelength irradiation and defect conditions, the 532 nm design is more sensitive to mismatched wavelengths.

The enhancement factor (*EF*) under mismatched incidence is defined as Equation (6).

(6) $$EF=\frac{E_{\max\left(mismatched\right)}-E_{\max\left(matched\right)}}{E_{max(matched)}}$$

*EF* applied throughout the analysis to quantify the relative change in *E*_max_ values between mismatched and matched conditions.

For G1 (shallow, narrow defects), the diagonal “matched” combinations (355/355, 532/532, and 1064/1064) are 1.35257/1.26231/1.21766, respectively. When irradiating a 532 nm AR with 355 nm incident light, EF increases by 11.1% over the matched value (1.35257) in the same row. The optimal *E*_max_ for 532 nm incident light occurs at the 1064 nm AR (1.28529), EF increases by 1.82% over the matched value (1.26231) in the same row. At 1064 nm incident light, the matched value (1.21766) is optimal, while the mismatch decreases (for example, the value is 1.13983 with a 355 nm AR, EF decrease by 6.39% over the matched value). This indicates that for G1, the 355 → 532 mismatch is the most critical, while the remaining conditions are close to or below the matched level.

For G2 (wide, shallow defects), the mismatch amplification effect is stronger, with the most dramatic fluctuations. The three “matched” diagonal lines are 1.35588/1.28395/1.20048, but within the same incident line, the mismatch can significantly raise the peak value: the AR of 355 nm incident with 532 nm reaches 1.63442, EF increases by 20.54% over the matched value (1.35588) in the same row; the AR of 532 nm incident with 355 nm is 1.46881, EF increases by 14.40% over the matched value; and the AR of 1064 nm incident with 532 nm is 1.39026, EF increases by 15.81% over the matched value. From a “coating-based wavelength” perspective, the 532 nm AR is particularly vulnerable in this geometry: when switching from a 532 nm matching (1.28395) to a 355 nm incident (1.63442), *E*_max_ surges by 27.30%. Similarly, the 1064 nm AR at 355 nm incident (1.45592), EF increases by 21.28% compared to its matching (1.20048). This is consistent with the “red-orange ridge” in the 3D plot, indicating that wide, shallow scratches, under coating-wavelength mismatch, form a large coherent enhancement band, a scenario most likely to trigger hotspot overflow.

For G3 (deep, narrow defects), a different mechanism emerges: the three “matched” combinations represent the maximum values for each row, while mismatches within the same row are generally lower than the match. For example, at 532 nm incident light, the AR values for 355/1064 nm are 1.49398/1.45480, respectively, both lower than 1.50929. Column-wise analysis also reveals that the “matched” AR values for 355 and 532 nm are both higher than the average values for different incident light sources (EF increases by approximately 4.35% and 12.96%, respectively), while the 1064 nm AR value actually increases slightly for mismatched incident light sources (with the average value being 7.54% higher than the match). This suggests that deep, narrow scratches are primarily driven by a strong localized resonance/squeezing effect induced by geometric constraints. When the coating layer and the incident wavelength are well matched, energy is more easily “locked” near the tip and interface, resulting in a more concentrated hotspot. However, mismatch weakens this geometry-driven energy convergence.

**Figure 6:**
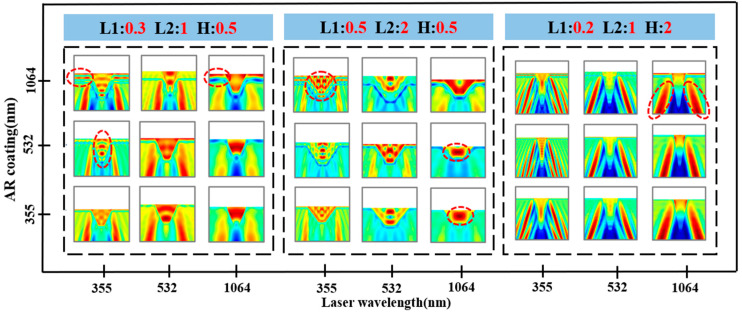
Electric field distribution maps of typical scratch geometries under multi-wavelength laser irradiation and AR coatings. The red dotted circle in the figure is the area with abnormally enhanced electric field, red is the area with enhanced electric field, and blue is the area with weakened electric field.

Figure 6 shows the corresponding electric field distribution. Overall, significant nonuniformity can be observed within the coating layer, a phenomenon closely related to the defect geometry and the coating’s design wavelength. When the incident wavelength matches the design wavelength of the AR coating, the lateral distribution of the electric field within the surface coating layer is relatively uniform, with hotspots primarily confined to the interior or tip of the defect. However, under mismatched conditions, a distinct striped interference pattern often appears within the coating layer, accompanied by multiple hotspots. This indicates that the lateral scattering introduced by the defect and the phase delay due to the coating thickness interact to cause multiple interferences within the coating layer and localized field enhancement.

In G1, when the incident laser wavelength matches the coating layer, the electric field intensity within the coating layer varies relatively smoothly, with little change in intensity within the defect. However, when 355 nm laser light is incident on the 532 nm and 1064 nm coatings, distinct lateral enhancement bands appear within the coating. In particular, during the transition from 355 nm to 1064 nm, the hotspot evolves from a single point to a band-like distribution, demonstrating the typical interference enhancement effect of short-wavelength incident light on long-wavelength coatings. When a 1064 nm laser is incident on the mismatched coating, the hotspot becomes less pronounced, primarily appearing within the defect and remaining a single hotspot. However, when irradiated on the matched coating, distinct enhancements and weakenings form within the coating.

In contrast, the wide and shallow scratch geometry of G2 more easily induces transverse mode coupling within the coating, resulting in a more complex hotspot evolution in the electric field distribution. When incident on a 355 nm laser, the number of hotspots gradually increases from a single central peak to a continuous fringe structure as the coating’s design wavelength increases (355 → 532 → 1064 nm). This indicates that as the optical path mismatch in the coating increases, the superposition effect of multiple reflections intensifies, leading to a more dispersed energy distribution within the coating and a wider hotspot region. At 1064 nm, when the coating’s design wavelength is mismatched, the hotspot converges to a single, strong peak within the defect, suppressing the fringe interference signature. When the coating’s wavelength is aligned, the central hotspot begins to disperse and forms a lateral enhancement on the coating surface. This is consistent with numerical statistical results, showing that G2 reaches its global maximum electric field intensity at a mismatch of 355 nm to 532 nm. This indicates that G2 exhibits the greatest risk when a short wavelength is incident on a long wavelength coating, while a relatively stable single hotspot distribution is observed with long wavelength incident light.

For deep, narrow scratches like G3, due to their large aspect ratio, the hotspot is almost always stably located near the defect sidewalls and extends toward the substrate, regardless of whether the incident wavelength matches the coating wavelength. A distinct electric field weakening point appears at the base of the defect tip and extends toward the substrate. The lateral distribution across the coating is relatively uniform, unlike the lateral streaks observed in shallow, narrow or wide, shallow defects. This indicates that the electric field distribution in deep, narrow scratches is primarily influenced by geometric cavity effects. The number of hotspots does not vary significantly with wavelength combinations. When the incident laser wavelength matches the coating wavelength, the streak intensity increases, and the maximum electric field intensity is also the highest.

The results in Figure 6 indicate that the uneven distribution of the electric field within the coating is determined by the three factors of defect geometry, coating dispersion, and incident wavelength. In shallow, narrow defects, the number and distribution of hot spots are particularly sensitive to the coating design wavelength. In wide, shallow defects, short-wavelength incident light on a long-wavelength coating induces the strongest multi-hot spot striping effect. In deep, narrow defects, hot spots are consistently concentrated near the defect sidewalls on the substrate and are less likely to migrate. However, the contrast of the interference fringes within the coating increases with increasing mismatch. This means that wide and shallow scratches pose the highest risk of component damage under multi-wavelength mismatch conditions, while deep and narrow scratches manifest as a “stable but dangerous” localized high field effect on the sidewalls, requiring special attention to the matching of coating layer design and defect control process.

Despite the insights gained, this study still has some limitations. First, we only simulated three representative scratch geometries. Although these geometries are typical, they cannot fully capture the various defect morphologies commonly found in actual optical components, such as pits and nodules. Second, the coatings and defects were idealized during modeling: the anti-reflection coating was assumed to follow a perfect quarter-wavelength design, the interface was defect-free, and the defect shape was simplified to a symmetrical trapezoidal profile. In reality, manufacturing defects and coating layer defects may further complicate the local field response. Third, this study is limited to FDTD-based electric field simulations and does not couple subsequent thermomechanical processes such as heat diffusion, stress accumulation, or crack growth, which are critical for linking field enhancement to actual damage evolution.

## 4. Discussion

The results reveal the coupling effects of AR coating design, incident wavelength, and defect geometry on nanoscale electric field modulation in fused silica. In the defect-free case, a quarter-wavelength double-layer AR coating was shown to effectively suppress the interface field at its design wavelength. Under matched conditions, the electric field distribution at the air–coating–substrate interface remains smooth, with minimal local enhancement and reduced penetration into the substrate. In contrast, when irradiated with an off-design wavelength, the coating exhibits significant interference fringes and field distribution distortion. Notably, the deviation increases with spectral detuning between the incident laser and the coating’s design wavelength, demonstrating that coatings optimized for a single wavelength cannot guarantee uniform suppression across multiple wavelengths. This observation is crucial for high-power systems with the coexistence of harmonics and parasitic wavelengths, as irradiation at off-design wavelengths can accumulate, significantly increasing the risk of damage.

When common defects are introduced, the interaction between the defect geometry and the AR coating further amplifies the field inhomogeneity. Shallow, narrow defects exhibit relatively confined perturbations, resulting in a single localized hotspot under matched irradiation, but evolve into a broader, band-like enhancement when a short-wavelength laser irradiates a long-wavelength coating. Wide, shallow defects are most sensitive to coating wavelength mismatch, producing multiple hotspots and extensive lateral, high-field fringes within the coating. In particular, the combination of 355 nm incident light and a 532 nm AR coating produces a global maximum enhancement factor of 1.63442, nearly 90% higher than the defect-free reference case, indicating that this condition is most susceptible to premature damage initiation. In contrast, deep, narrow defects, regardless of mismatch, consistently concentrate the field near the defect sidewalls and produce highly localized, needle-like hotspots due to waveguiding and confinement effects. Although mismatch increases fringe contrast within the coating, the hotspot location remains fixed within the substrate, indicating that geometry dominates the localization mechanism.

Another key observation is that under mismatch conditions, the field distribution within the AR coating is inhomogeneous. Even in defect-free samples, detuned wavelengths produce lateral field fringes within the coating; these inhomogeneities are significantly amplified in the presence of defects. For wide, shallow geometries, the mismatch not only produces stronger hot spots but also multiple interference bands across the coating thickness, whereas for deep, narrow geometries, coating interference enhances defect sidewall localization. These results suggest that AR coatings inherently smooth the interface field at the target wavelength, but their effectiveness decreases in the presence of spectral detuning. Defects transform these modest distortions into critical, localized enhancements whose severity depends on the geometry. These findings emphasize the importance of considering both defect geometry and coating dispersion to mitigate damage in high-power, multi-wavelength laser systems.

Our results also provide valuable insights for the design of AR coatings for broadband applications. The design of AR coatings for broadband applications must consider the effects of the coating layers under different wavelengths of incidence. Because wavelength mismatch in AR coatings for single-wavelength applications leads to significant field enhancement, using only a two-layer AR coating is insufficient to fully accommodate multiple wavelengths. Designing multilayer AR coatings may be an effective strategy for optimizing for multiple wavelengths. By simulating the amplified electric field distribution within the substrate under multi-wavelength incidence, the thickness, material, and arrangement order of each AR coating layer can be effectively selected to achieve optimal results.

Despite the insights gained, this study has several limitations. First, we simulated only three representative scratch geometries. While these geometries are representative, they cannot fully capture the diverse defect morphologies commonly found in real optical components, such as pits and nodules. The coatings and defects were idealized during modeling: the antireflection coating was assumed to follow a perfect quarter-wavelength design, the interface was defect-free, and the defect shape was simplified to a symmetrical trapezoidal profile. In reality, manufacturing and coating defects can further complicate the local field response. Second, the FDTD model is based on continuous-wave and nanosecond-scale laser simulations and does not fully capture the electric field intensity distribution characteristics under laser irradiation at shorter time scales and higher peak powers, such as time-dependent phenomena associated with laser irradiation at the femtosecond scale. These phenomena are potential future developments. Third, this study is limited to FDTD-based electric field simulations and does not incorporate subsequent thermomechanical processes, such as heat diffusion, stress accumulation, or crack growth, which are crucial for linking field enhancement to actual damage evolution.

## 5. Conclusions

This study systematically investigated the electric field modulation of a double-layer AR-coated fused silica optical component under multi-wavelength laser irradiation using finite-difference time-domain simulation. The results show that, under defect-free conditions, an AR coating designed with a quarter-wavelength thickness effectively suppresses the interface field at the target wavelength, resulting in a smooth field distribution and achieving the desired AR effect. However, under off-design irradiation conditions, interference fringes and field distortion appear within the coating layer. Furthermore, the substrate field intensity increases with increasing spectral detuning, highlighting the risks posed by harmonic and off-design wavelength laser irradiation in high-power laser systems.

When representative surface defects are introduced, the coupling between geometry, coating dispersion, and incident wavelength further amplifies the field inhomogeneity. Shallow and narrow defects induce relatively small perturbations that widen under mismatch conditions. Wide and shallow defects, however, exhibit the greatest mismatch sensitivity, generating multiple hot spots and transverse high-field fringes within the coating. For a 532 nm AR coating with an incident wavelength of 355 nm, the global maximum enhancement factor is 1.63442, nearly 90% higher than the defect-free reference. In contrast, deep, narrow defects further enhance the field and confine it to the defect sidewalls close to the substrate, generating needle-shaped hotspots dominated by confinement and waveguiding effects.

Taken together, these findings demonstrate that coating dispersion and defect geometry have a decisive influence on the nanoscale electric field distribution in fused silica under multi-wavelength irradiation. While AR coatings can attenuate interfacial fields at the designed wavelength, they cannot prevent distortions under detuned conditions, where defects transform these modest distortions into critical localized enhancements. This work provides fundamental quantitative insights into the defect-coating-wavelength coupling mechanism and offers valuable guidance for the design of defect-tolerant AR coatings, predictive lifetime assessments, and the development of nanostructured, optimized optical components for next-generation high-power laser systems.

## Figures and Tables

**Table 1:** Quarter-wave thickness and refractive index parameters of the three sets of double-layer anti-reflection coatings at their respective design wavelengths.

Design Wavelength (nm)	Layer Type	Material	Quarter-Wave Thickness t (nm)	Refractive Index *n*	Extinction Coefficient k
355	coatings	SiO_2_	60	1.4760	~1 × 10^−6^
		HfO_2_	43.3	2.1804	~1 × 10^−4^
	substrate	Fused Silica	-	1.4760	~1 × 10^−6^
532	coatings	SiO_2_	91.2	1.4608	~1 × 10^−6^
		Ta_2_O_5_	60.8	2.1501	~1 × 10^−4^
	substrate	Fused Silica	-	1.4607	~1 × 10^−6^
1064	coatings	SiO_2_	182.4	1.4497	~1 × 10^−6^
		Ta_2_O_5_	126.7	2.0801	~1 × 10^−5^
	substrate	Fused Silica	-	1.4496	~1 × 10^−6^

**Table 2:** *E*_max_ of fused silica with different scratch geometries, AR coatings, and incident laser wavelengths.

	L1: 0.3 μm, L2: 1 μm, H: 0.5 μm			L1: 0.5 μm, L2: 2 μm, H: 0.5 μm			L1: 0.2 μm, L2: 1 μm, H: 2 μm		
	355	532	1064	355	532	1064	355	532	1064
355 nm	1.35257	1.50213	1.38774	1.35588	1.63442	1.45592	1.45300	1.36475	1.40696
532 nm	1.23902	1.26231	1.28529	1.46881	1.28395	1.44810	1.49398	1.50929	1.45480
1064 nm	1.13983	1.18038	1.21766	1.30203	1.39026	1.20048	1.28566	1.26250	1.33056

## Data Availability

The authors confirm that the data supporting the findings of this study are available within the article.
